# Comprehensive analysis of cuproptosis-related genes in diabetic cardiomyopathy

**DOI:** 10.1371/journal.pone.0328512

**Published:** 2025-10-27

**Authors:** Jun Li, Hua Zhang, Xinyue Duan, Meina Zhang, Xin Li, Chunyan Hao

**Affiliations:** 1 First Clinical Medical College of Shanxi Medical University, Taiyuan, Shanxi, China; 2 Second Clinical Medical College of Shanxi Medical University, Taiyuan, Shanxi, China; 3 Department of Gerontology, First Hospital of Shanxi Medical University, Taiyuan, Shanxi, China; The Hong Kong University of Science and Technology, HONG KONG

## Abstract

**Background:**

Diabetic cardiomyopathy (DCM) represents a distinct myocardial pathology arising from chronic diabetic metabolic disturbances, characterized by progressive structural and functional abnormalities that frequently culminate in heart failure. Cuproptosis, a novel form of cell death, is highly linked to mitochondrial metabolism and mediated by protein lipoylation. However, studies are limited on the clinical significance of cuproptosis-related genes (CRGs) in DCM. Therefore, it is helpful to identify CRGs involved in DCM and explore their expression and molecular mechanisms.

**Methods:**

We downloaded three datasets of DCM from the GEO database and a set of cuproptosis-related genes with 176 genes. Following the identification of the differentially expressed cuproptosis-related genes(DECRGs) and hub genes, we performed the functional annotation, protein-protein interaction network, co-expression network analysis, mRNA-miRNA regulatory network.The GSE5606 dataset was then used to verify hub genes. Finally, we validated the expression of hub genes in a high glucose-induced H9C2 cell injury model via RT-qPCR.

**Results:**

We identified 14 DEFRGs and 7 key genes in the DCM samples compared to the control. Functional enrichment analysis revealed that DECRGs are associated with several pathways, including TCA cycle, respiratory electron transport, oxidative stress, and metabolism pathway. Moreover, Isocitrate dehydrogenase 1(Idh1), Cytochrome P450 Family 1 Subfamily A Member 1(Cyp1a1), 3-Hydroxy-3-Methylglutaryl-CoA Synthase (Hmgcs2) and Hexokinase 2(Hk2) were identified as the hub genes and validated in the GSE5606 datasets with area under the curve(AUC)>0.7. The qRT-PCR results showed that the expression levels of Cyp1a1, Hmgcs2, HK2, and Idh1 in vitro model of DCM and controls were consistent with the bioinformatics analysis results from the mRNA microarray.

**Conclusions:**

Overall,we identified hub genes and pathways involved in DCM via bioinformatics analysis and revealed the potential role of cuproptosis, providing useful and novel information to explore the potential candidate genes for DCM diagnostic and therapeutic options.

## Introduction

Diabetes, as a global chronic disease among millions of people, is caused by the inability to produce insulin because of pancreatic dysfunction (type 1 diabetes) or caused by the malfunction of cells to use insulin (type 2 diabetes) [1,2]. Diabetic cardiomyopathy (DCM) is one of the major complications of type 1 and type 2 diabetes [3,4], accompanied by altered cardiac energetics, impaired mitochondrial function, chronic inflammatory responses, and oxidative stress, manifested as heart failure in the absence of coronary artery disease, hypertension, valvular heart disease, and congenital cardiac anomalies [5,6]. However, early detection of diabetic cardiomyopathy has proven to be difficult in a clinical setting. Although the underlying mechanisms have been extensively investigated [7], the exact molecular mechanism and pathways associated with DCM are still far from being elucidated, and there is no direct and effective prevention and treatment for DCM [8,9].

Copper is an essential micronutrient used by cells for critical biological processes [10,11].Copper homeostasis is crucial for maintaining various physiological functions in organisms [12,13], such as mitochondrial energy production [14], redox homeostasis [15], and extracellular matrix remodeling, but it can produce cytotoxicity reaction when the content of copper ions in cells exceeds the threshold for maintaining homeostasis [16]. Cellular copper overload activates the apoptosis pathway via endogenous and exogenous pathways [17,18]. In addition, copper overload also leads to cuproptosis, a newly proposed RCD pattern, mediates cell death mainly through cytotoxicity caused by the mitochondria-dependent increase in energy metabolism and accumulation of reactive oxygen species (ROS) [19], and this pattern has been shown to be closely related to the tricarboxylic acid cycle (TCA).This copper-induced cell death is characterized by, on the one hand, the decrease in lipoylation of DLAT and DLST and the oligomerization of lipoylated proteins of the TCA cycle induced by the direct copper binding and, on the other hand, destabilization and overall reduction in iron-sulfur (Fe-S) cluster proteins, which together lead to proteotoxic stress and mitochondrial dysfunction [20].

Research indicates that the levels of copper in scalp hair and bloodstream are markedly elevated in individuals with diabetes compared to non-diabetic controls [21]. Cuproptosis, driven by oxidative stress and mitochondrial dysfunction, while diabetes alters cardiomyocyte metabolism, also leading to oxidative stress that may lead to increased copper levels within cells [22]. Copper promotes ROS generation, insulin resistance, and diabetes occurrence, leading to the development of atherosclerosis [23]. Moreover, accumulation of catalytically active Cu^2+^ in the cardiac extracellular myocardial induces copper toxicity, which is proposed as an important catalyst of cardiovascular damage in diabetes [24]. Copper toxicity leads to protein oxidation, glutathione(GSH) depletion, lipid damage, and redox imbalance [25,26], which could cause impairment of heart function. In addition, cardiomyocytes have the highest number of mitochondria.Therefore, it is reasonable to assume the potential induction of cuproptosis, which is expected to become another unique angle to explore the pathogenesis of DCM.

Hence, the purpose of this article is to identify the cuproptosis mechanisms and central gene of diabetic cardiomyopathy via bioinformatic analysis and, more significantly, to verify the expression of hub cuproptosis-related genes in vitro model of DCM by RT-qPCR.

## Materials and methods

### Datasets collection

We used the keyword “diabetic heart or diabetic cardiomyopathy” to search the microarray or RNA-seq datasets from the GEO database of NCBI (https://www.ncbi.nlm.nih.gov/). Finally, four gene expression profiles (GSE4745, GSE6880, GSE5606, GSE267352) were obtained from Gene Expression Omnibus (GEO). GSE4745 dataset was based on the GPL85 platform, [RG_U34A] Affymetrix Rat Genome U34 Array done by Gerber et al. It contains 24 samples of the ventricular gene array data of Rattus norvegicus, including 12 controls and 12 diabetics induced by streptozotocin (STZ) injection. To better analyze the differential genes between control (CON) group and DCM group, 8 samples collected on day 42, including 4 DCM samples and 4 CON samples, were selected for analysis [27]. The GSE6880 ( [RAE230A] Afymetrix Rat Expression 230A Array) is generated by the GPL341 platform comprising 6 LV samples from CON rats (N = 3) and DCM rats (N = 3) [28].The GSE5606 dataset was used as the validation dataset and generated using GPL1355 platform and composed of 14 LV samples from CON rats (N = 7) and DCM rats (N = 7) [29]. The RNA-seq GSE267352 (Illumina NovaSeq 6000) is generated by the GPL25947 platform comprising 6 heart samples from CON rats (N = 3) and DCM rats (N = 3) [30]. This dataset is employed to assess the robustness and reproducibility of the GSE4745 and GSE6880 gene expression profiles. A total of 176 cuproptosis-related genes(CRGs) were obtained from GeneCards (www.genecards.org) and previously published articles [20,31,32]. The list of cuproptosis-related genes with source information is provided in the S1 File.

### Differential expression analysis

GEO2R (https://www.ncbi.nlm.nih.gov/geo/geo2r/), an interactive web tool, performs differential expression analysis utilizing the Limma package and GEOquery R libraries. Quantile normalization and volcano maps were generated by using the “ggplot2” package in the R software. The screening threshold for significant diferentially expressed genes(DEGs) was adjusted with |log2FC| ≥ 0.2 and *adj.P < *0.05. A Venn diagram was created using the online tool Venny 2.1.0 (http://bioinfogp.cnb.csic.es/tools/venny/index.html) [33] to identify the CoDEGs (upregulated and downregulated genes) in GSE4745 and GSE6880.

### Identifcation of diferentially expressed cuproptosis-related genes

The CoDEGs(upregulated and downregulated genes) in GSE4745 and GSE6880 were intersected with the cuproptosis related genes(CRGs) to obtain differentially expressed cuproptosis-related genes (DECRGs). Based on the raw sequencing data in GSE4745 and GSE6880, heatmaps of DECRGs were generated by employing the ComplexHeatmap package [2.13.1] in the R software [34].

### Functional and pathway enrichment analysis

The DAVID(Database for Annotation, Visualization, and Integrated Discovery) was used to perform Gene Ontology (GO) analysis and Kyoto Encyclopedia of Genes and Genomes (KEGG) pathway enrichment analysis of DECRGs [35]. Differentially expressed genes(DEGs) from the GSE267352 were used for KEGG analysis. GO was composed of cell components (CC), biological processes (BP) and molecular function (MF). Gene expression patterns were visualized using the ggplot2 package (v3.4.0) in R (v4.2.1), with statistical significance defined as *P* < 0.05. Gene Set Enrichment Analysis (GSEA) was conducted utilizing the WebGestalt platform [36].

### Protein–protein interaction (PPI) network construction

The PPI network of the DECRGs was constructed according to the information acquired using the STRING database(https://string-db.org/) with the condition that the interaction combined score was > 0.4 points. Molecular interaction networks of DECRGs were constructed and visualized using Cytoscape (version 3.8.1).

### Hub gene selection and co-expression analyses

Core regulatory genes within the DECRGs were identified through topological analysis using CytoHubba, a Cytoscape plugin, which evaluates node centrality in protein-protein interaction networks. A subset of five centrality algorithms (Closeness, Degree, EPC, MCC, MNC) was systematically selected from CytoHubba’s twelve available methods. The intersection of top-ranked genes (n = 8) across these algorithms was used to identify high-confidence hub genes. Subsequently, a co-expression network of hub genes was constructed using GeneMANIA(http://www.genemania.org) [37], an established web-based platform for functional gene analysis and interaction network prediction

### Construction of the mRNA–miRNA regulatory network

TargetScan (version 6.2) is a credible database that mainly centers on miRNA-target interactions. Potential miRNAs targeting the hub genes were predicted using TargetScan (v8.0), with subsequent validation required through either independent database confirmation or experimental evidence. Finally,we constructed and visualized the mRNA-miRNA regulatory network utilizing Cytoscape [38].

### Validation of hub genes in GSE5606

The microarray GSE5606 served as the independent dataset for validation purposes.The expression data of 7 hub genes were extracted, and groups were compared using the Student’s t-test. To evaluate the diagnostic performance of 7 hub genes, the ROC was plotted using the“pROC”R package. The statistical significance was set at **P* *< 0.05.

### H9C2 cell culture and establishment of vitro model of DCM

H9C2 rat cardiomyocytes were purchased from Wuhan Pricella Biotechnology Co., Ltd. H9c2 cells were cultured in DMEM medium containing 10% FBS, 100 U/ml penicillin and 100 μg/ml streptomycin in 5%CO2,37°C, and passaged or punched when cells grew to about 80% to 90% confluence state for experiments. H9c2 cells were grown in complete medium containing 50 mmol/L glucose DMEM for 24 h to establish an in vitro DCM model [39].

### Quantitative real-time polymerase chain reaction

According to the manufacturer’s protocol, total RNA was isolated using M5 Universal RNA Mini Kit (Mei5bio, Beijing, China), and then synthesized with Hifair® III 1st Strand cDNA Synthesis SuperMix for qPCR (Yeasen, Shanghai, China). Real-time polymerase chain reaction (PCR) was performed using Hieff UNICON® Universal Blue qPCR SYBR Green Master Mix (Yeasen, Shanghai, China) via the LightCycler® 96 Instrument. β-tublin was used as the reference gene. All gene expressions were analyzed using the comparative Ct method (2^-∆∆Ct). Details of all primers sequences were shown in Table 1. Independent sample T test was used to compare the control group and DCM group. CRGs with *P* < 0.05 was considered statistically significant.

**Table 1:** Primers for real-time polymerase chain reaction.

Gene	Forward primer	Reverse primer
Cyp1a1	CAATGAGTTTGGGGAGGTTACTG	CCCTTCTCAAATGTCCTGTAGTG
Hmgcs2	TTGCCCTGGAGGTCTATTTTCC	GAAGCCCATACGGGTCTGG
Hk2	CACGGCGAGTTTGGTTCCTA	CTTCTGGCTTTGGGCGTAGT
Idh1	ACAAGTCCCAGTTTGAAGCTC	GCACATCCCCATCGTAATTCTTA
Txnrd1	GGGTCCTATGACTTCGACCTG	AGTCGGTGTGACAAAATCCAAG
Nfe2l2	CTGAACTCCTGGACGGGACTA	CGGTGGGTCTCCGTAAATGG
Dlst	CTCGGCACAAGGATGCTTTC	GGGCCTTCTCTCCTAGTTCATTA

## Results

### Identification of DEGs and differentially expressed cupr-optosis-related genes(DECRGs)

**Fig 1:**
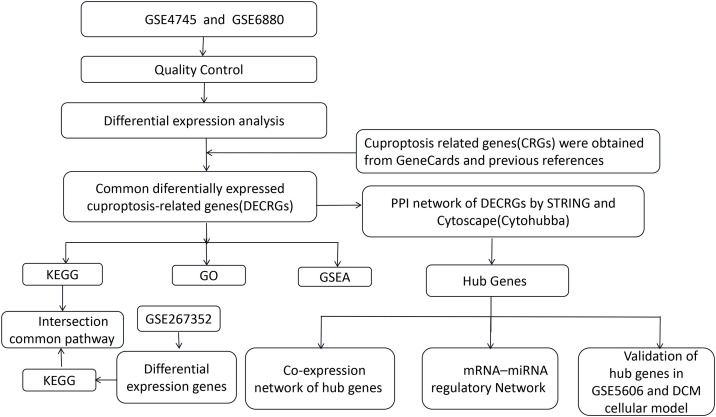
Flow chart of methodologies applied in the study.

**Fig 2:**
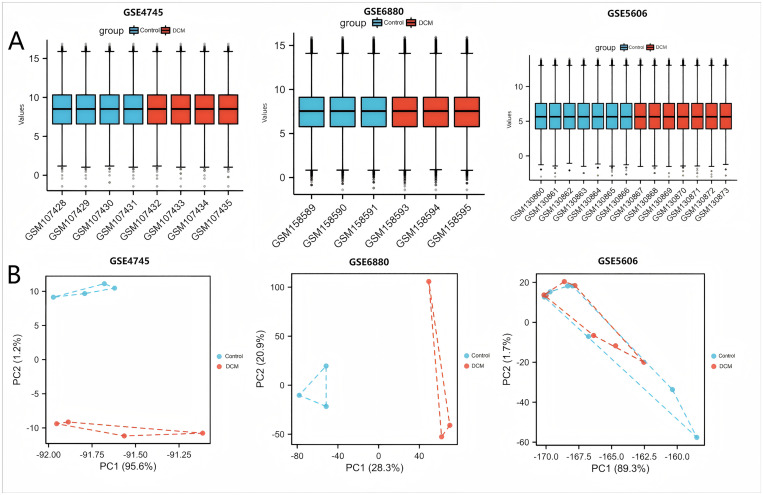
Data Distribution and Sample Clustering. (A) Box plots of standardized GSE4745, GSE6880 and GSE5606. The x-axis label represents the sample symbol and the y-axis label represents the gene expression values. (B) PCA plots of DCM and healthy control group in GSE4745, GSE6880 and GSE5606. PCA,principal component analysis.

**Fig 3:**
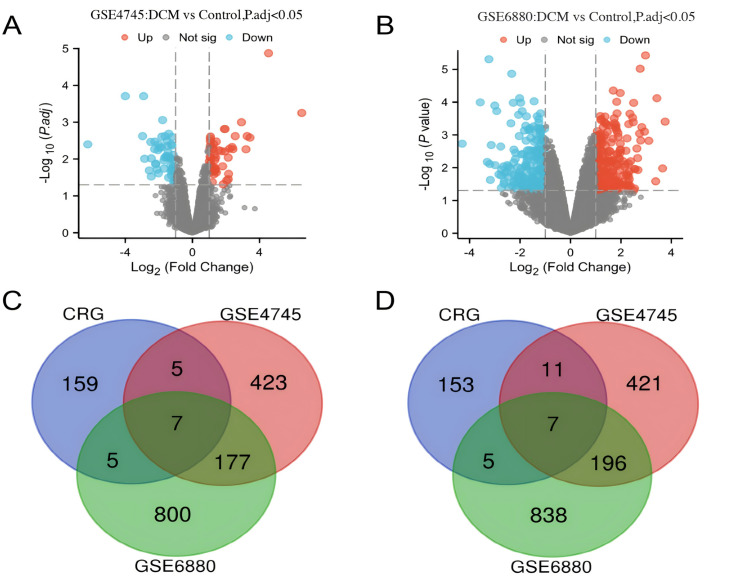
Identification of differentially expressed cuproptosis-related genes (C-RGs) in GSE4745 and GSE6880. (A)Volcano plots of DEGs in GSE4745. (B)Volcano plots of DEGs in GSE6880. Blue dots indicate the downregulated genes, while red dots indicate the upregulated genes. DEGs, differentially expressed genes. (C)Venn diagram of up-regulated DEGs in GSE4745, GSE6880, and CRGs. (D)Venn diagram of down-regulated DEGs in GSE4745, GSE6880, and CRGs.

**Fig 4:**
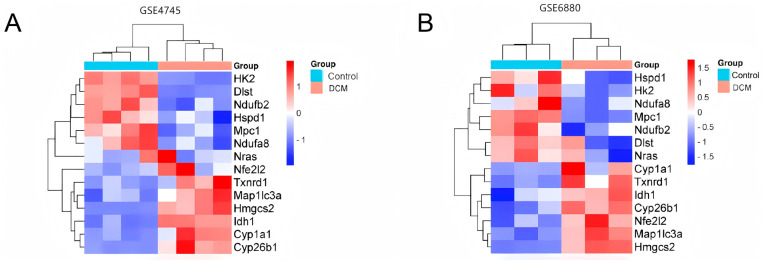
Heatmap for the DECRGs. (A) Heatmap of DECRGs identified in GSE4745. (B) Heatmap of DECRGs identified in GSE6880. DECRGs, differentially expressed cuproptosis-related genes.

The schematic overview of the research design is presented in Fig 1. Standard uniformity and comparability of the gene expression in each sample in the dataset were reflected by box plots (Fig 2A, S1 Fig). Principal component analysis (PCA) revealed significant sample clustering patterns across all datasets, demonstrating clear biological differentiation (Fig 2B). The volcano plots of these DEGs in GSE4745 and GSE6880 are shown in Figs 3A and 3B. After standardization and identification of the microarray results, there were 1562 DEGs in GSE4745 (773 upregulated and 789 downregulated genes) and 2196 DEGs in GSE6880 (1063 upregulated and 1133 downregulated genes). Then, we intersected DEGs with 176 CRGs and obtained 14 DECRGs, including 7 upregulated and 7 downregulated genes (Figs 3C and 3D). The clustered heatmaps of DECRGs in the GSE4745 and GSE6880 datasets are shown in Figs 4A and 4B.

### Functional enrichment analysis of differentially expressed genes

**Fig 5:**
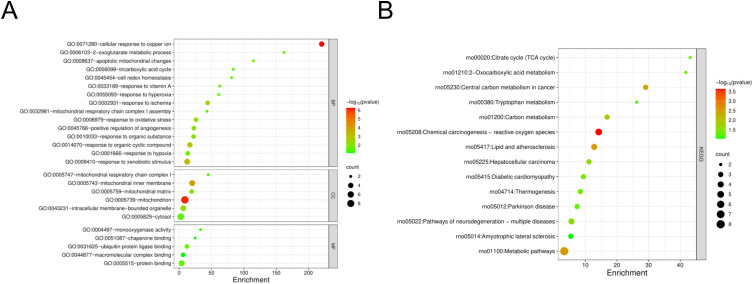
GO and KEGG enrichment analysis of DECRGs. (A) GO enrichment analysis of the DECRGs. (B) KEGG enrichment analysis of the DECRGs.

**Fig 6:**
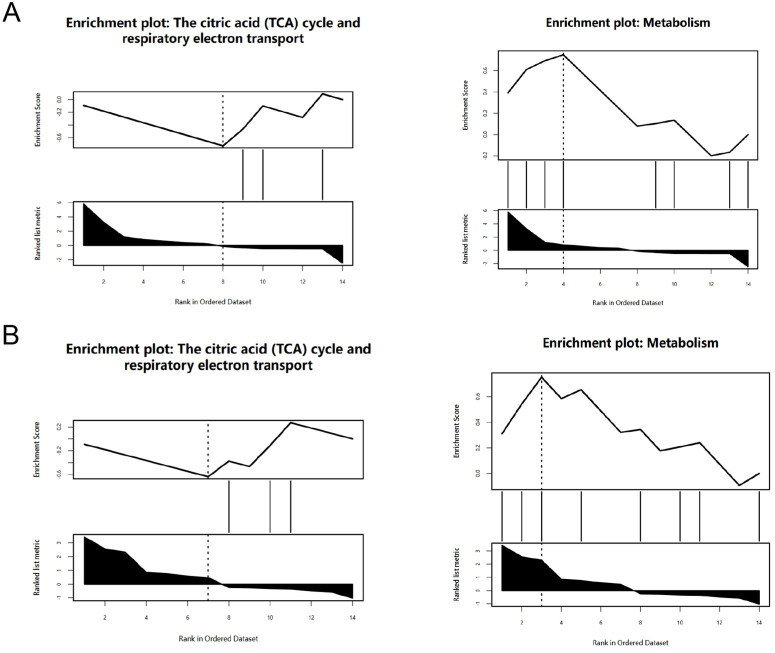
GSEA enrichment analysis. (A) The enrichment pathway analysis of DECRGs in GSE4745. (B) The enrichment pathway analysis of DECRGs in GSE6880.

The DAVID was used for GO and KEGG enrichment analyses of DECRGs. Gene Ontology (GO) enrichment results were categorized into three functional domains: biological processes (BP), cellular components (CC), and molecular functions (MF) (Fig 5A). The BP was mainly enriched in cellular response to copper ion, response to ischemia, response to oxidative stress, response to hypoxia, response to organic cyclic compound, response to xenobiotic stimulus, tricarboxylic acid(TCA) cycle. The CC comprised the mitochondrion, cytosol, mitochondrial matrix, mitochondrial inner membrane, intracellular membrane-bounded organelle. Furthermore, the MF analysis demonstrated significant enrichment of protein-binding activities, including macromolecular complex binding, ubiquitin-protein ligase interactions, and chaperone binding. Significant KEGG pathway enrichment analysis of DECRGs revealed critical involvement of metabolic pathways, chemical carcinogenesis-reactive oxygen species, lipid and atherosclerosis, diabetic cardiomyopathy carbon metabolism, TCA cycle, 2-Oxocarboxylic acid metabolism, Parkinson disease, amyotrophic lateral sclerosis, thermogenesis (Fig 5B). KEGG enrichment analysis of DEGs in GSE267352 primarily enrichment in chemical carcinogenesis-reactive oxygen species, diabetic cardiomyopathy, amyotrophic lateral sclerosis, carbon metabolism, TCA cycle, Parkinson disease, amyotrophic lateral sclerosis, thermogenesis, Alzheimer disease, Huntington disease, 2-Oxocarboxylic acid metabolism(S2 Fig). The intersection of the two KEGG enrichment results identifies 8 common pathways(S1 Table). GSEA revealed significant enrichment of metabolic pathways, particularly those associated with the TCA cycle and mitochondrial electron transport chain(Figs 6A and 6B).

### Protein-protein interaction network construction

**Fig 7:**
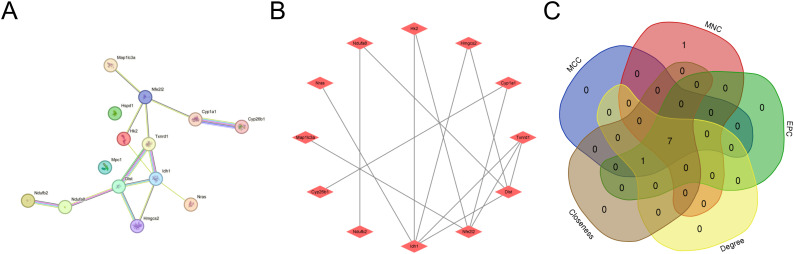
PPI network and identification of hub genes. (A) PPI network of the DECRGs was generated using the STRING database. (B) PPI network of the DECRGs constructed by Cytoscape software. (C) Seven hub genes were identified from the intersection of top-ranked candidates across five cytoHubba algorithms.

Based on STRING database predictions (minimum confidence threshold = 0.4), we established a DECRG interaction network that, when visualized through Cytoscape, exhibited a modular structure containing 14 protein nodes and 14 interaction edges(Figs 7A and 7B). The interaction network comprised an equal distribution of DECRGs, with 7 upregulated and 7 downregulated members, demonstrating significant interconnectivity among most network components.

### Hub gene selection and co-expression analysis

**Fig 8:**
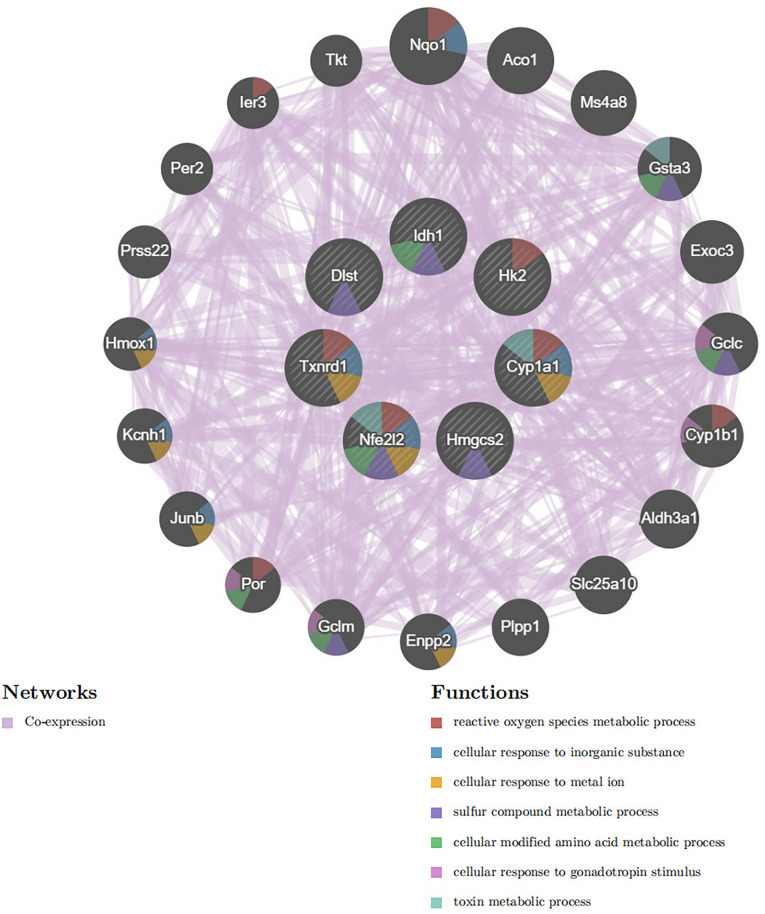
Hub genes and their co-expression genes were analyzed using GeneMANIA.

In this article, five algorithms (Closeness, Degree, EPC, MCC, MNC) were adopted in CytoHubba to identify hub genes. The consensus genes identified across all five algorithms (top 8 candidates) are presented in Table 2. The intersection of the top 8 candidate genes derived from the five algorithms was subsequently analyzed to identify core regulatory genes (Fig 7C). Five upregulated genes(Idh1, Cyp1a1, Nfe2l2, Hmgcs2, Txnrd1) and two down-regulated genes(Hk2, Dlst) were screened as pivotal genes (Table 3). Subsequently, we employed the GeneMANIA platform to construct and analyze an interaction network comprising the 7 hub genes and their co-expressed partners (Fig 8).

**Table 2:** Top-ranked hub genes identified by 5 cytoHubba algorithms.

MCC	MNC	EPC	Degree	Closeness
Idh1	Dlst	Idh1	Idh1	Idh1
Dlst	Idh1	Dlst	Nfe2l2	Dlst
Nfe2l2	Hmgcs2	Txnrd1	Dlst	Nfe2l2
Txnrd1	Txnrd1	Nfe2l2	Txnrd1	Txnrd1
Hmgcs2	Hk2	Hk2	Hmgcs2	Hk2
Hk2	Cyp1a1	Hmgcs2	Hk2	Hmgcs2
Cyp1a1	Nfe2l2	Ndufa8	Cyp1a1	Ndufa8
Ndufa8	Nras	Cyp1a1	Ndufa8	Cyp1a1

**Table 3:** The seven hub genes include 5 upregulated and 2 downregulated genes in DCM.

Gene	Description	DCM
Cyp1a1	Cytochrome P450 Family 1 Subfamily A Member 1	UP
Hmgcs2	3-Hydroxy-3-Methylglutaryl-CoA Synthase 2	UP
Hk2	Hexokinase 2	DOWN
Idh1	Isocitrate dehydrogenase 1	UP
Txnrd1	Thioredoxin Reductase 1	UP
Nfe2l2	NFE2 Like BZIP Transcription Factor 2	UP
Dlst	Dihydrolipoamide S-Succinyltransferase	DOWN

### Construction of mRNA-miRNA regulatory network

**Fig 9:**
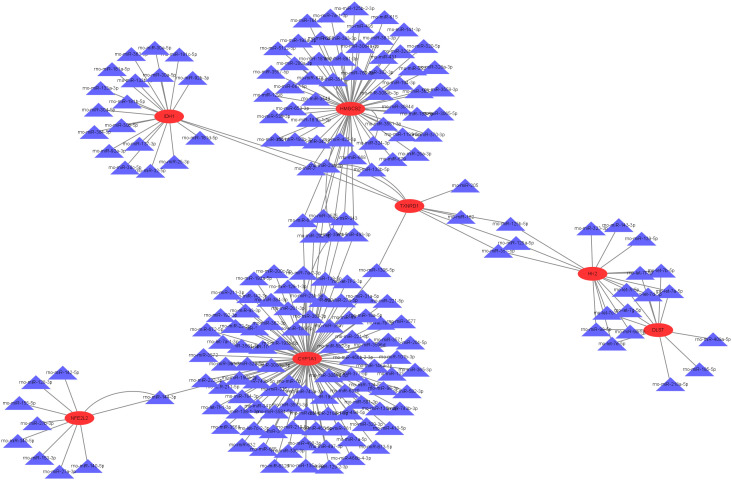
A hub gene mRNA-miRNA regulatory network was modeled in Cytoscape.

Bioinformatic analysis of hub genes via TargetScan yielded 231 miRNAs meeting stringent criteria requiring experimental validation and database confirmation. Subsequently, a comprehensive regulatory network integrating hub genes and their predicted miRNA interactors was constructed and visualized using Cytoscape(Fig 9). The constructed network offers insights into potential regulatory relationships and functional interactions among core genes.

### Validation of hub genes expression and the diagnostic value in GSE5606

**Fig 10:**
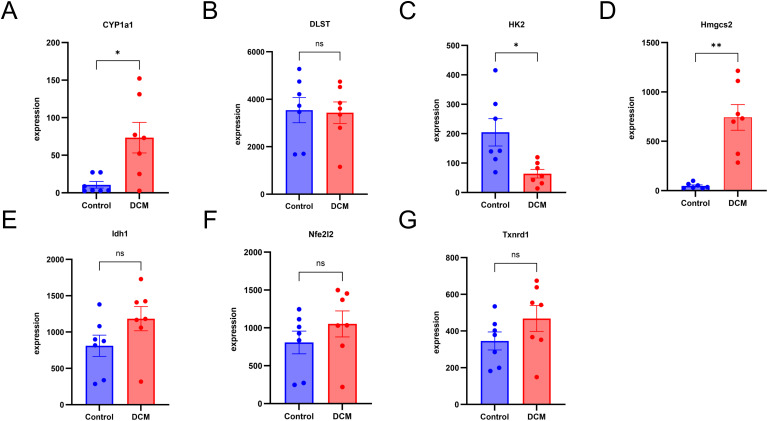
Validation of hub genes in dataset GSE5606. (A) Cyp1-a1. (B) Dlst. (C) Hk2. (D) Hmgcs2. (E) Idh1.(F) Nfe2l2. (G) Txnrd1. Sample, N_control_ = 7, N_DCM_ = 7. Data are presented as mean±standard error of the mean (SEM). *P < 0.05; **P < 0.01; ns, not significant.

To validate the robustness of our bioinformatics findings, we performed independent validation using dataset GSE5606, confirming the expression patterns of the seven hub genes in DCM samples. We found that the expression of Cyp1a1 and Hmgcs2 was significantly upregulated in the DCM samples, the expression of Hk2 was significantly downregulated in the DCM samples, Idh1, Dlst and Txnrd1 Nfe2l2 did not reach statistical significance (Fig 10, S2 File). Although Idh1 did not reach statistical significance, its expression levels in DCM cardiac tissue were higher than in non-diseased samples.

**Fig 11:**
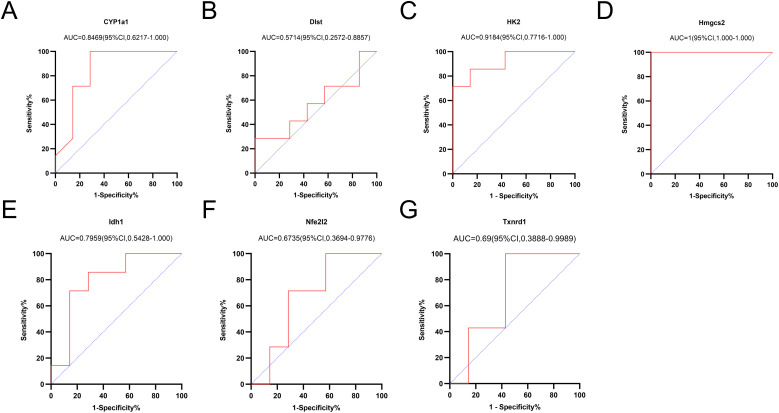
The diagnostic values of hub genes in DCM in the GSE5606 by ROC analysis. (A) Cyp1-a1. (B) Dlst. (C) Hk2. (D) Hmgcs2. (E) Idh1.(F) Nfe2l2. (G) Txnrd1. Sample, N_control_ = 7, N_DCM_ = 7.

The area under curve (AUC) of Cyp1a1, Hmgcs2, Hk2, Idh1, Txnrd1, Nfe2l2 and Dlst for DCM was 0.847, 1.0, 0.918, 0.796, 0.694, 0.673,0.571 respectively in the GSE5606 dateset, with Cyp1a1, Hmgcs2, HK2 and Idh1 demonstrating good diagnostic value (Fig 11). Analysis of comprehensive expression level and diagnostic value, Cyp1a1, Hmgcs2, Hk2 and Idh1 were identified as the core regulatory genes.

### Hub gene expression verification in vitro model of DCM

**Fig 12:**
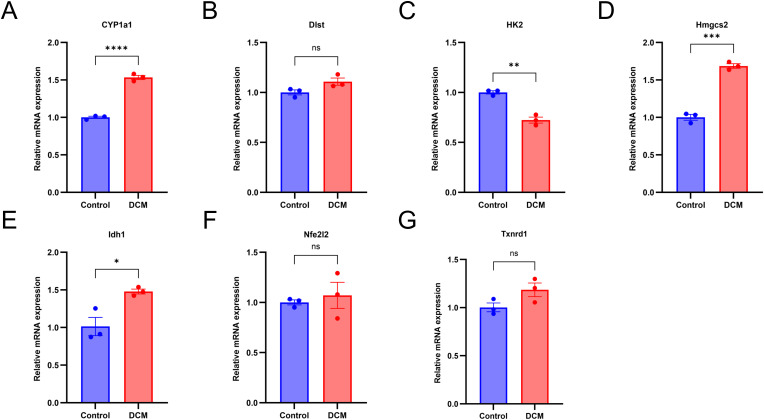
Expression of CRGs in H9C2 DCM model. (A) Cyp1a1. (B) Dlst. (C) Hk2. (D) Hmgcs2. (E) Idh1.(F) Nfe2l2. (G) Txnrd1. Changes in mRNA expression of hub genes by qRT-PCR (n = 3). Data are presented as mean±standard error of the mean (SEM). *P < 0.05; **P < 0.01; ***P < 0.001; ****P < 0.0001; ns, not significant.

The expression of 7 hub genes were further verified to study the effect of cuproptosis on diabetic cardiomyopathy.The results showed 4 of them were differentially expressed(Fig 12, S3 File).They were Cyp1a1 (up),Hmgcs2 (up),IDH1 (up) and HK2 (down).The expression of Cyp1a1, Hmgcs2, IDH1 and HK2 were consistent with the predicted result.

## Discussion

Through integrative analysis of two GEO datasets and CRGs, we identified 14 DECRGs, comprising 7 upregulated and 7 downregulated genes. Comprehensive functional annotation of DECRGs was performed to delineate their putative biological roles and pathway associations. Gene Ontology (GO) enrichment analysis revealed significant localization of DECRG-encoded proteins to mitochondrial compartments, including the inner membrane,matrix, and respiratory chain complex I, along with cytosolic and membrane-bounded organelle distributions; they are involved in cellular response to copper ion, response to ischemia and hypoxia, response to oxidative stress, TCA cycle, implying that DECRGs might partake in the initiation and progression of DCM through these biological processes. These two KEGG enrichment analyses reveal a significant overlap of biological pathways, which to some extent demonstrates the robustness and reproducibility of the GSE4745 and GSE6880. KEGG enrichment analysis revealed that carbon metabolism, oxidative stress, and TCA cycle were significant pathway, which may be associated with DCM development. Besides, GSEA demonstrated that most of the DECRGs were mainly enriched in the TCA cycle and respiratory electron transport and metabolism pathway, which have collectively been confirmed as essential mechanisms in cuproptosis, thus contributing to the pathogenesis of DCM. We constructed the PPI network and screened 7 hub genes(Idh1, Dlst, Cyp1a1, Hk2, Nfe2l2, Hmgcs2, Txnrd1), all of which are potential candidate biomarkers closely associated to DCM. Integrated functional annotation and co-expression network analysis implicated reactive oxygen species (ROS) metabolism, metal ion responsiveness, sulfur compound biogenesis, and modified amino acid metabolism in the molecular pathogenesis of cuproptosis during DCM progression. Next, we validated the hub candidate genes in dataset GSE5606 and vitro model of DCM, Cyp1a1, Hmgcs2, Hk2 and Idh1 were consistent with bioinformatic predictions.

Based on the TargetScan database predictions of seven candidate genes, a total of 231 miRNAs were obtained with the condition that the predicted miRNA could be verified by other databases.Subsequently, a regulatory network of candidate genes and predicted miRNAs was generated by Cytoscape software. However, this research did not experimentally validate the regulatory interactions between the candidate genes and the predicted microRNAs. Given the complex regulatory network of microRNAs—where one microRNA can target multiple mRNAs and one mRNA can be regulated by multiple microRNAs—along with their spatiotemporal expression patterns in specific cell types and pathological conditions, experimental validation remains a challenging endeavor. Moreover, empirical validation generally demands significant temporal investment, financial resources, and specialized experimental parameters (such as suitable cellular or animal models), which are presently beyond the scope of our current research capabilities. Further experimental procedures in this investigation include transfecting cells within an in vitro diabetic cardiomyopathy model using miRNA mimics for gene overexpression or inhibitors for gene silencing to elucidate their functional significance. This is subsequently complemented by dual-luciferase reporter assays in HEK293T cells to verify the specific miRNA-mRNA interaction sites.

As a cytochrome P450 monooxygenase, Cyp1a1 initially observed in the endoplasmic reticulum, and then found to be localized on the mitochondrial inner membrane [40,41]. Mitochondria are abundant in mammalian cardiomyocytes. Disturbances in mitochondrial metabolism can significantly affect cardiomyopathy. Chen et al. [42] found that Cyp1a1 mRNA expressions in STZ induced diabetic mice heart ventricles was 12-fold higher than that in the control group. Moreover, Cyp1a1 expression is negatively correlated with ejection fraction,fractional shortening and posterior wall thickness in diabetic mice, whereas they showed the opposite results in non-diabetic mice. Our experimental results also show that the expression of Cyp1a1 in the DCM model group is higher than that in the control group, suggesting that Cyp1a1 may be involved in the occurrence of DCM. The copper content in scalp hair and blood is significantly higher in diabetic patients compared to those without diabetes [21], copper ions lead to glutathione (GSH) depletion by covalently binding to protein sulfhydryl groups, which increases ROS generation and subsequently induces the expression of the gene Cyp1a1 through the negative regulation of the NF-κB signaling pathway [43]. Additionally, copper ions also induce the expression of the gene Cyp1a1 by activating the AP-1 signaling pathway [43].Overexpression of Cyp1a1 can produce excessive ROS which in turn promotes fatty acid peroxidation and inhibits the activity of mitochondrial respiratory chain, leading to myocardial injuries [40].Therefore, Cyp1a1 may influence the pathogenesis and progression of DCM through cuproptosis.

Hmgcs2 (3-Hydroxy-3-Methylglutaryl-CoA Synthase 2) is distributed to the mitochondrial matrix and is a crucial enzyme that limits ketone synthesis [44]. The expression and activity of HMGCS2 (3-hydroxy-3-methylglutaryl-CoA synthase 2) are regulated by multiple transcription factors as well as post-translational modifications, including acetylation [45]. The activity of ketone body synthetase HMGCS2 was significantly increased in the heart of db/db mouse, indicating the possible involvement of Hmgcs2 in DCM [46]. Another study revealed that high expression of PPARα and HMGCS2 in DCM, and PPARα silencing could decrease the expression of HMGCS2, thereby alleviating the myocardial injury and oxidative stress in DCM rats [47]. In addtion, our in vitro study also confirmed that high glucose (HG) induces the upregulation of HMGCS2 expression in cardiac myocytes..Xie et al.‘s study [48] found that knocking out the hepatic ceruloplasmin gene in high-fat diet-fed mice restored hepatic copper levels. This led to increased intracellular copper binding to SCO1, which subsequently enhanced the expression of mitochondrial fatty acid oxidation (FAO) genes (including HMGCS2 and Cpt1α) by activating the AMPK-PGC1α-PPARα axis., implying that HMGCS2 may play a regulatory role in the process of cuproptosis.

Hk2 (Hexokinase 2) is an isozyme that catalyzes glucose phosphorylation and functions as a rate-limiting enzyme in the aerobic glycolytic pathway. HK2 is mainly expressed in myocardium, where it functions as an important role in myocardial protection [49]. By positioning itself on the outer membrane of mitochondria, the formation of voltage-dependent anion channel(VDAC)-1 and HK2 complex modulated the integrity and permeability of mitochondria membrane [50]. Cuproptosis is closely associated with mitochondrial damage and disrupted metabolic pathways, in the process of cuproptosis, Cu^2+^ combines with the lipoylated components of the tricarboxylic acid cycle in the mitochondrial respiratory chain, resulting in the oligomerization of lipoylated protein, followed by proteotoxic stress as well as cell death [20]. With the reduction of HK2 in mitochondria, the interaction between HK2 and VDAC-1 was impaired, which resulted in dramatic increase of mitochondria membrane permeability and release of pro-apoptotic proteins and cytochrome C, thereby triggering apoptosis [50]. Moreover, HK2 dissociation from mitochondria promotes oligomerization of VDAC that facilitates inflammasome activation [51]. Additionally, HK2 deficiency results in a significant reduction in glycolytic flux and thus cells are adapting by increasing basal respiration and mitochondrial efficiency, hence at a steady-state condition, a slightly but not significantly higher TCA flux can generate a better proton-motive force in the mitochondria, allowing electron transport chain energy to be transferred more efficiently to ADP, thereby compensatorily upregulating mitochondrial oxidative phosphorylation (OXPHOS) [52]. The inactivation of HK2 redirects glycolytic flux toward mitochondrial metabolism, which is considered vital for increasing cellular sensitivity to cuproptosis (cells that rely more on mitochondrial respiration are nearly 1000 fold more sensitive to Cu^+^ than cells that primarily rely on glycolysis) [50,53]. These studies suggest a potential connection between cuproptosis and HK2.

The isocitrate dehydrogenase (IDH) enzyme family comprises three distinct isoforms (IDH1, IDH2, and IDH3) that serve as critical catalytic components in the TCA cycle [54].To investigate the impact of IDH mutations on cardiac remodeling, researchers generated mice bearing hematopoietic cells with an Idh2R140Q mutation [55], which mimics one of the most common IDH1/2 mutations in acute myeloid leukemia (AML) patients. IDH mutations lead to increased production and accumulation of the oncometabolite D-2-hydroxyglutarate (D2-HG) through a neomorphic enzymatic function. By inhibiting α-ketoglutarate dehydrogenase (α-KGDH) and ATP synthase, D2-HG disrupts the tricarboxylic acid (TCA) cycle, reduces mitochondrial membrane potential (MMP), and increases reactive oxygen species (ROS) generation. These alterations directly impair cardiac energy metabolism and drive epigenetic remodeling, ultimately resulting in heart failure. Therefore, IDH1 may be associated with cuproptosis.The role of IDH1 in diabetic cardiomyopathy has been scarcely studied, and further investigations are still required in the future.

There are some limitations in this study. First, we acknowledge that relatively old microarray datasets (GSE4745, GSE6880 and GSE5606) utilized in this study, while extensively validated in prior literature [46,56,57], may limit the biological relevance and clinical translatability of the findings, particularly given the advances in RNA-seq technology and improved annotation in recent years. However, our focus on core cuproptosis genes in DCM benefits from the robust within-platform consistency of these datasets. Future studies integrating multi-platform data would further strengthen these findings. Second, our bioinformatics approach relied exclusively on publicly available datasets, which may not fully represent human biological variability due to the absence of dedicated clinical samples.

## Conclusions

In summary, this study used bioinformatics methods to identify hub cuproptosis-related genes (Cyp1a1,Hmgcs2,Hk2 and Idh1) and pathways involved in DCM and revealed the potential role of cuproptosis. The ROC curve demonstrated their significant predictive value. Furthermore, the validation in H9C2 cells under high glucose conditions confirmed the 4 hub genes. Although further research is still needed, we provide useful and novel information to explore the potential candidate genes for DCM diagnostic and therapeutic options. The follow-up work in our lab is to testify the effect of those hub genes deletion or overexpression on the progress of DCM, and to investigate the potential molecular mechanisms using transgenic mice.

## Supporting information

S1 Fig Box plots of the gene expression data before normalization. (TIF)

S2 Fig KEGG enrichment analysis of the DEGs in GSE267352. (TIF)

S1 Table 8 common pathways identified by intersecting the results of two KEGG analyses. (DOCX)

S1 File 176 cuproptosis-related genes with source information. (XLSX)

S2 File The expression profiles of 7 hub genes in GSE5606. (XLSX)

S3 File Q-PCR expression analysis of 7 hub genes. (XLSX)
