# Supplementary figures and images for: Comprehensive analysis of cuproptosis-related genes in diabetic cardiomyopathy
Source: PLoS One. 2025 Oct 27;20(10):e0328512. doi: 10.1371/journal.pone.0328512 (PMC12558461; doi:10.1371/journal.pone.0328512)

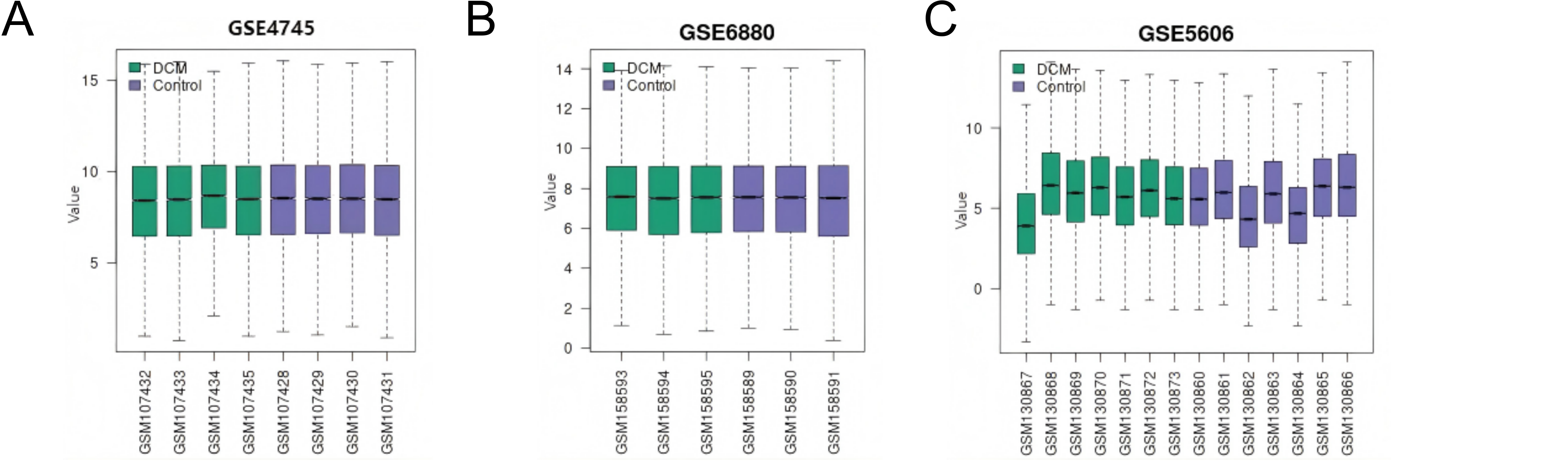

Supplement: S1 Fig — (TIF) [file pone.0328512.s001.tif]

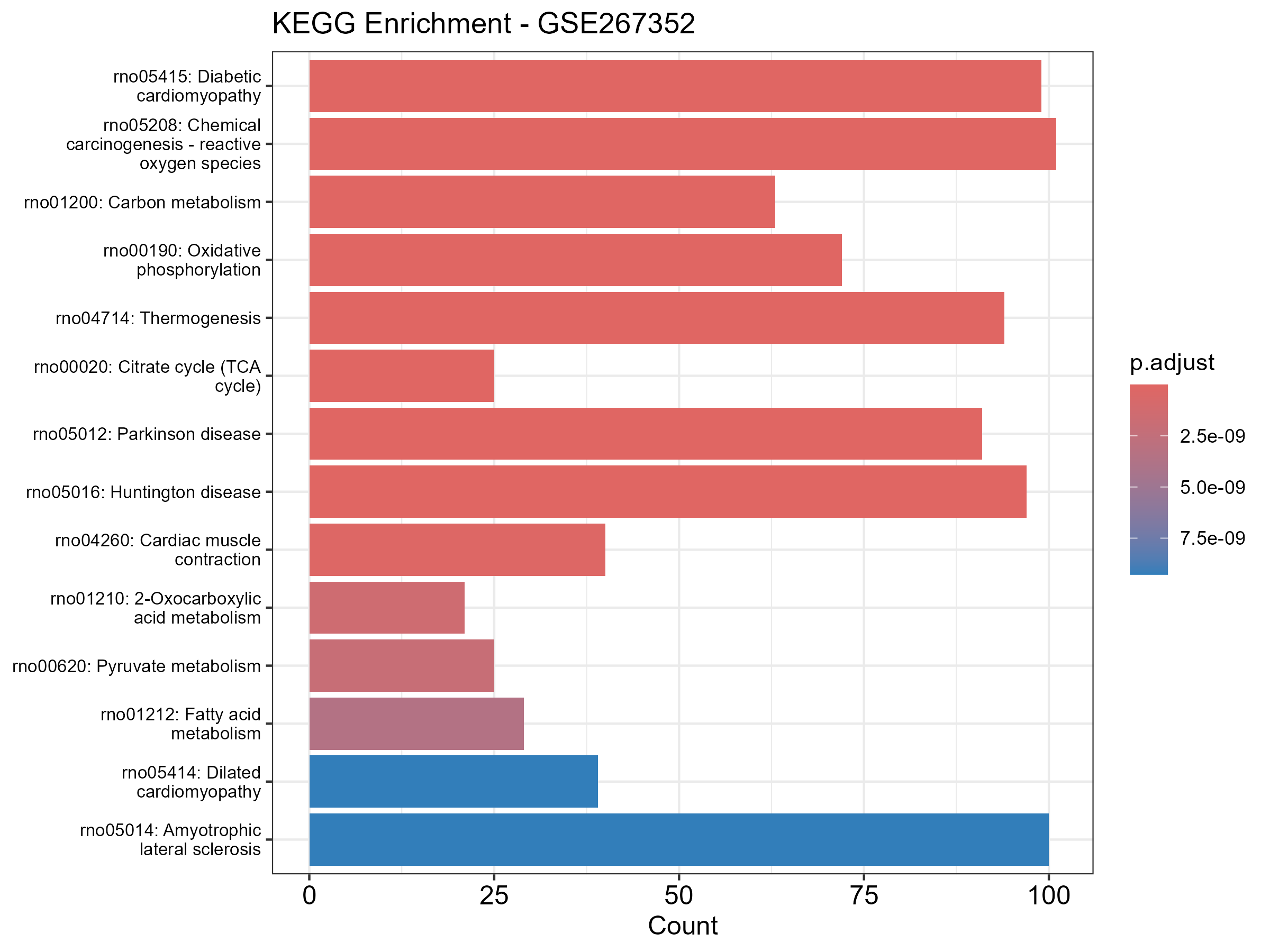

Supplement: S2 Fig — (TIF) [file pone.0328512.s002.tif]
