# Supplementary material for: Comprehensive analysis of cuproptosis-related genes in diabetic cardiomyopathy
Source: PLoS One. 2025 Oct 27;20(10):e0328512. doi: 10.1371/journal.pone.0328512 (PMC12558461; doi:10.1371/journal.pone.0328512)
Supplement: S1 Table — (DOCX) [file pone.0328512.s003.docx]

**S1 Table.** **8 common pathways identified by intersecting the results of two KEGG analyses**

| **ID** | **Term** |
| --- | --- |
| rno00020 | TCA cycle |
| rno05208 | Chemical carcinogenesis-reactive oxygen species |
| rno05415 | Diabetic cardiomyopathy |
| rno01200 | Carbon metabolism |
| rno04714 | Thermogenesis |
| rno01210 | 2-Oxocarboxylic acid metabolism |
| rno05014 | Amyotrophic lateral sclerosis |
| rno05012 | Parkinson disease |
